# Recent Advances in Epsilon-Poly-L-Lysine and L-Lysine-Based Dendrimer Synthesis, Modification, and Biomedical Applications

**DOI:** 10.3389/fchem.2021.659304

**Published:** 2021-03-30

**Authors:** Sijin Chen, Shuting Huang, Yan Li, Chuncai Zhou

**Affiliations:** School of Material Science and Engineering, Tongji University, Shanghai, China

**Keywords:** polypeptide, ε-poly-L-lysine, lysine-based dendrimers, amphiphilic coploymer, therapeutic application

## Abstract

With the advantages in biocompatibility, antimicrobial ability, and comparative facile synthesis technology, poly-L-lysine (PLL) has received considerable attention in recent years. Different arrangement forms and structures of the backbone endow lysine-based polymers with versatile applications, especially for ε-poly-L-lysine (EPL) and lysine-based dendrimer (LBD) compounds. This review summarized the advanced development of the synthesis and modification strategies of EPL and LBD, focus on the modification of bio-synthesis and artificial synthesis, respectively. Meanwhile, biomedical fields, where EPL and LBD are mainly utilized, such as agents, adjuvants, or carriers to anti-pathogen or used in tumor or gene therapies, are also introduced. With the deeper of knowledge of pharmacodynamics and pharmacokinetics of the drug system, the design and synthesis of these drugs can be further optimized. Furthermore, the performances of combination with other advanced methodologies and technologies demonstrated that challenges, such as scale production and high expenses, will not hinder the prospective future of lysine-based polymers.

**Graphical Abstract:**
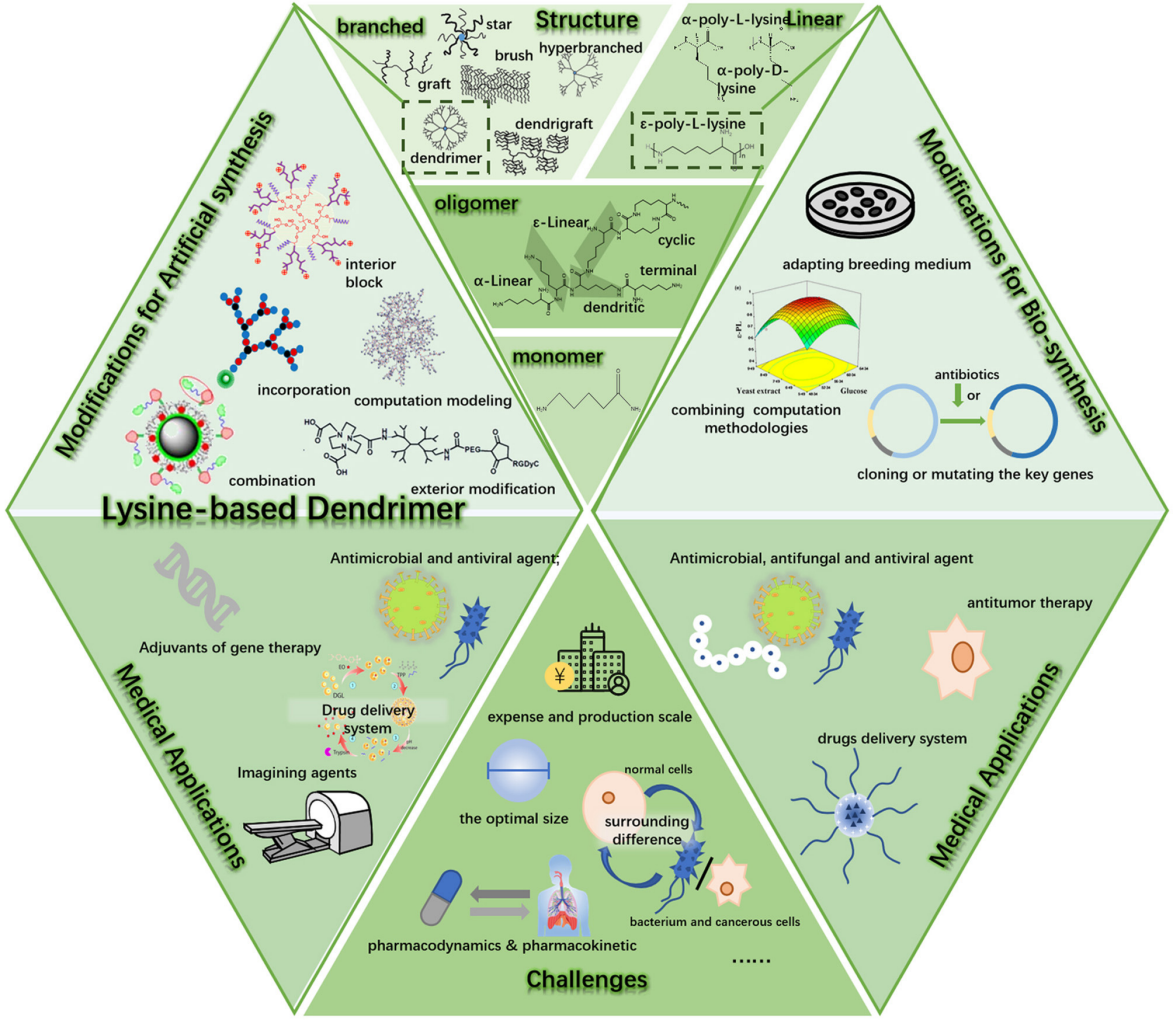
Synthesis, modification, and biomedical applications of EPL and LBD.

## Introduction

**Figure 1:**
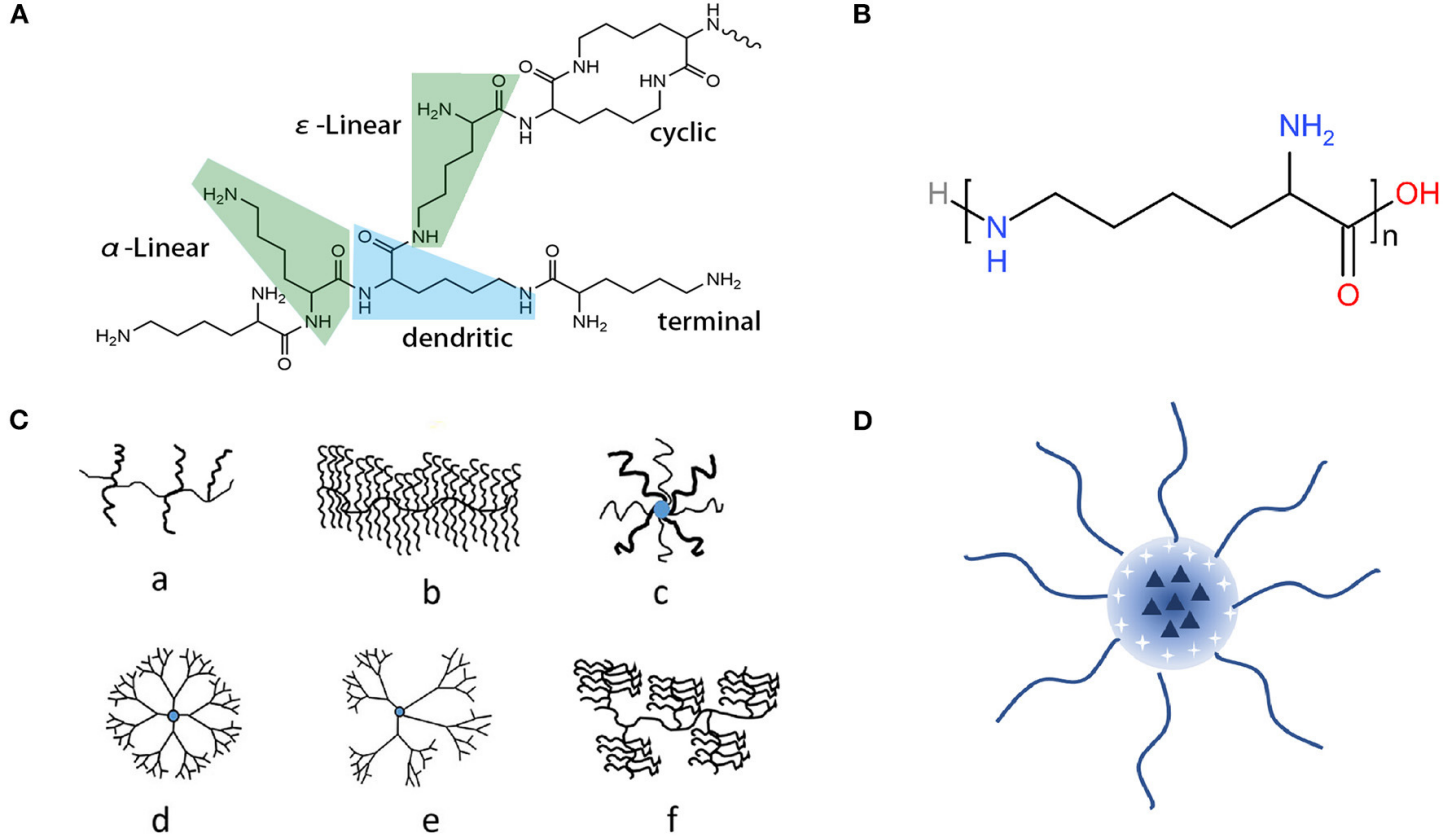
Structures of PLL-related polymers with different morphologies. **(A)** The molecular structure of PLL (Ge et al., 2019); **(B)** The structure of EPL, one of the linear PLL forms; **(C)** Different molecular structure polylysine derivatives (Shi et al., 2015): (a) graft, (b) brush, (c) star architectures, (d) dendrimer, (e) hyperbranched, and (f) dendrigraft; **(D)** Self-assembled nanoparticles of PLL derivatives (Li Y. et al., 2020).

With great biocompatibility and controllable yet numerous compositions, polyamino-acid, which consists of several kinds of amino acid residues, exhibits attractive properties (Graphical Abstract). Among different types of polyamino-acids, poly-L-lysine (PLL) has earned considerable attention in recent years, showing great performances in several areas, not only including areas like food preservation (Tuersuntuoheti et al., 2019) and environmental pollution treatment (Kwon et al., 2017; Bucatariu et al., 2020) but also been widely used in medical applications (Shukla et al., 2012; Shi et al., 2015; Rodrigues et al., 2020). The structure of these kinds of polymers could vary, including cycle, dendritic, α-linear, and ε-linear (Figure 1A). While their chain structure could not only be formed as linear (Figure 1B), but also branched, like graft, brush, star architecture, dendrimer, hyperbranched, and dendrigraft (Figure 1C). The PLL's structure could even be more complicated when conjugated with other polymers to form block copolymers (Wu T. et al., 2018), or they could self-assemble to micelles (Figure 1D) when modified as an amphipathic chain (Li et al., 2016). Different structures endow them with different applications. Among those related topics, the diverse modification strategies and versatile properties of linear ε-poly-L-lysine (EPL) and lysine-based dendrimer (LBD) stand out.

In accordance with their main chain structure, PLL can be classified into two types: α-poly-L-lysine and EPL, with α- or ε-linear made up as their main backbone, respectively. EPL is a linear homopolyamide consisting of 25–35 L-lysine residues with linkages, between the α-carboxyl and ε-amino group of adjacent residues (Shi et al., 2015). Since its first separation from nature in 1977 (Shima and Sakai, 1977), the structure, synthesis, and modification strategies, together with numerous explorations for a broader application of EPL have been remarkably researched in recent years. EPL shows much better antimicrobial activity and selectivity than its isomeride, such as α-PLL and α-poly-D-lysine (Mayandi et al., 2020). Advantages, such as water solubility, excellent thermal stability, and eco-friendliness (Liang et al., 2014; Fursatz et al., 2018; Jiang et al., 2019), provide EPL with great convenience and potential to be processed facilely. Up to now, it has established a fairly mature scale production path. EPL could also be used in modifying other drugs and improving their compatibility and affinity to human tissues due to its great biocompatibility. Native defensive activity also stimulates the use of EPL as an antimicrobial agent. Remarkable abilities, such as edible, biocompatible, and broad antimicrobial spectrum, widen its application to fields, including food protection (Tuersuntuoheti et al., 2019), agriculture (Jiang et al., 2019; Rodrigues et al., 2020), and biomedical agents. Therefore, exploring the processing techniques and applications of EPL has attracted many concerns nowadays.

In addition to the linear structure, another attractive structure of PLL belongs to the dendritic part. Since its first synthesis in 1985 (Tomalia et al., 1985), studies focusing on dendrimer structure have poured in, and this structure showed better properties than the linear forms in terms of transfection efficiency due to its higher cationic density, which may also simultaneously increase cytotoxicity (Alazzo et al., 2019). Dendritic materials composed of different compounds have expanded in recent years, including poly(amidoamine), polyetherimide, and LBD (Francoia et al., 2017). Among them, LBD has gained a place for its great biodegradable and biocompatible ability (Wu C. et al., 2018). In the present review, the LBD not only contained the dendrimer that consisted only by lysine residue as PLL with a dendritic structure (Walsh et al., 2018, 2019) but also included those structures with PLL dendron matrix with some modifications. With an increase in molecular weight, the ability of LBD to penetrate membranes, such as the blood–brain barrier, may be weakened, while longer retention time and higher efficiency could be achieved as renal excretion and elimination decreasing (Zhang et al., 2017). In addition, a denser functional group on the surface provides LBD with substantial ability to conjugate with other drugs and larger possibilities to be used as drug carriers. Up to now, LBD has been widely used in studies of tumor treatments, including as a carrier or an adjuvant of some special therapies, such as positron emission tomography. Its conjugation alone could also be potentially used as an antimicrobial, antifungal or antiviral agent. Moreover, LBD could be used in cosmetic and supramolecular photocatalytic assembly (Attia-Vigneau et al., 2020; Tian et al., 2020), and more potential applications of this polymer could be identified in the future.

In this review, the synthesis strategies and diverse applications of EPL and LBD were summarized, including antimicrobial and antiviral agent, drug delivery system, adjuvants of imaging techniques combined with other compounds, and some related technologies incorporated in drug design and analysis process, such as digital simulation and metabonomic or proteomic studies. Some issues and breakpoints for future development were also identified. By addressing these problems, these two kinds of polymers shall have a brighter future and extensive applications in human lives.

## *ε*-Poly-L-Lysine

### Synthesis and Modification

**Figure 2:**
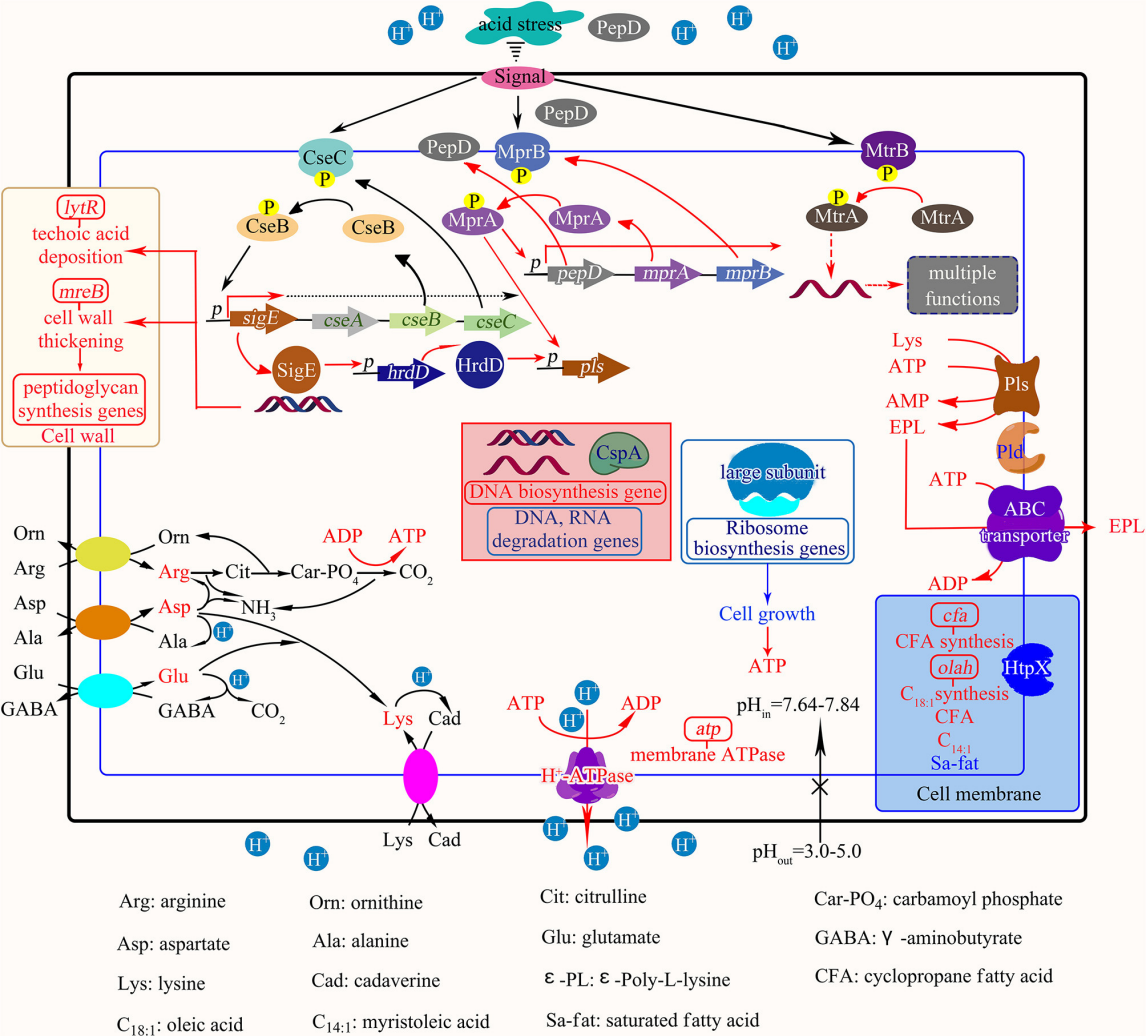
The physiological and transcriptional response mechanisms of *S. albulus* to spontaneous acid stress in the commercial biosynthesis of EPL (Wang C. Y. et al., 2020).

The two main strategies for EPL production are chemical synthesis and biosynthesis. With a similar constitution, the synthesis of α-PLL could simply transforms to EPL by replacing the activator system from dicyclohexyl carbodiimide and 18-crown-6 ether in chloroform to 1-ethyl-3-(3-dimethylaminopropyl) carbodiimide in aqueous medium (Ho et al., 2008). However, limitations, such as small-scale and complicated polymerization processes, hinder the extensive use of chemically synthesized EPL. EPL, as a natural existing product, its production mainly relies on biosynthesis with few kinds of microorganisms, such as *Streptomycetaceae, Kitasatospora sp*., and *Ergot fungi* (Shukla et al., 2012; Wang et al., 2019). Furthermore, the process of commercially producing EPL especially relies on *Streptomyces albulus* fermentation (Figure 2) as the metabolic process of this microbe combating acid stress (Wang C. Y. et al., 2020).

As the value of EPL has been proven with gradual in-depth exploration, larger yield is urgently needed. Modifications to the production of EPL are mainly in three ways: (i) adapting breeding medium by adding or by replacing. The former additives could be astaxanthin, which works as an antioxidant to reduce intracellular oxidant response and downregulated relevant gene transcription (Li S. et al., 2020), while the latter pointing to replacing the carbon sources to cyclodextrins and glycerol for producing low-molecular-weight EPL, which exhibits more powerful antimicrobial activity than its larger counterparts (Chen et al., 2018); (ii) optimizing the producing strategies by combining other computation methodologies. For example, researchers used artificial neural network modeling and surface response methodology to assist to the optimization of the medium composition then enlarging the yield (Bhattacharya et al., 2017; Guo et al., 2018); or (iii) cloning or mutating the key genes of those microorganism, activating a larger production of EPL (Liu et al., 2019; Wang et al., 2019; Xu et al., 2019). Furthermore, with a more precise production or synthesis mechanism, such as determining the opposite and negative elements that may influence the production, higher yield with a more time-saving and effective production process could be achieved, which could be the preferred pathway for further studies.

### Advanced Applications of EPL in Biopharmaceuticals

EPL is generally believed to be safe, and it has been permitted by the Food and Drug Administration in several counties as a commercial food preservative (Cai et al., 2015; Buzon-Duran et al., 2019; Chen et al., 2019; Wang C. Y. et al., 2020). With its excellent polycationic property related to its abundant amino terminals in physiological environment and the propensity to self-assemble when modified as an amphipathic molecular (Huang et al., 2015; Choi et al., 2018; Niu et al., 2019; Li Y. et al., 2020), EPL has become a great candidate to be used in numerous fields, including antimicrobial agents, drug delivery carriers, and adjuvants in biomedical fields.

#### Antimicrobial, Antifungal, and Antiviral Agents

EPL shows great antibacterial effect to several microorganisms, including Gram-positive and -negative bacteria, fungi, yeast, and bacteriophage, and could even effectively beat plant viral diseases (Chen et al., 2019; Jiang et al., 2019). By using this broad-spectrum inhibition activity, EPL could be used in suture or dressing material when conjugated with polyacrylic acid/polyvinyl acetate electro-spun nanofiber or polysaccharide compounds, such as gellan gum and bacterial cellulose (Amariei et al., 2018; Fursatz et al., 2018; Peng et al., 2020). They could not only maintain the mechanical properties of the matrix while endowing them with great antimicrobial ability, but also even exhibit antibiofilm ability without inducing inflammatory response in animal experiments. As the loading mass of EPL in the matrix may limit the performance of the antimicrobial ability of EPL (Amariei et al., 2018), the relationship and affecting mechanism between them deserve further studies. When EPL binds to carboxymethyl cellulose, self-degradable bacteriostatic hydrogels with rapid gelation and low irritation could be formed, with the potential for point embolization or as a medical hemostatic dressing (Wang Y. et al., 2020). Also, great water solubility and biodegradability provide EPL and its conjugations with better compatibility in physiological environment, thus cause lower toxic to host tissues (Yang et al., 2021). Meanwhile, EPL shows better antibacterial performance when combined with other water-soluble and biocompatible materials, such as chitosan oligomers with Maillard reaction (Liang et al., 2014; Buzon-Duran et al., 2019). This effect also brings the possibility of EPL to be used as a modifier or carrier for drugs with poor compatibility.

In spite of many advantages, EPL still shows some limitations, such as poor antioxidant property. At the same time, its strong polycationic property could bring great affinity to other anion materials in the culturing environment, which may affect its antibacterial ability. For the first problem, the Maillard reaction has been used to modify EPL with several monosaccharides (Zhang et al., 2019). The samples showed 40.5–69.4% diphenylpicryl phenylhydrazine scavenging activity after a prolong reaction time and exhibited a heat time-dependent increase in reducing activity with low cytotoxicity and a slight decrease in antimicrobial ability. For overcoming the potential electrostatic interaction with phosphate groups that attenuates the antimicrobial ability of EPL, Jiang's group (Jiang et al., 2019) synthesized a nanocomplex of EPL with deoxyribonucleic acid (DNA) by nanoprecipitation method. The nanoparticles showed a broad-spectrum inhibition, and could maintain the ability even in the adverse condition of phosphate system.

However, if the modification agent react with the terminal amino functional groups of EPL without provide other antibacterial groups, the antibacterial activity of the drug may be compromised. So, balance between the intrinsic antimicrobial activities and modification is indispensable in EPL-related antimicrobial drugs designing.

#### Adjuvants to Antitumor Therapy

Cancer has reportedly overtaken cardiovascular disease in recent years as the leading cause of death in developed countries (Mahase, 2019), and the cancer treatment strategies have plagued researchers for a long time. However, recent studies have demonstrated that EPL could play a role in cancer therapy, including in the invention of new antitumor drugs, imaging diagnostic adjunctive therapies, such as positron emission tomography, and gene therapy, thereby confirming the potential of EPL as an adjuvant for antitumor therapy.

Docetaxel (DOX) and curcumin are two main drugs normally used in cancer treatment, but their severe cytotoxicity without specificity hinders their applications (Xu et al., 2017; Li Y. et al., 2020). With excellent biocompatibility and several active sites in the side chain, EPL and its derivatives could be used as a good candidate for antitumor drug carrier system. Combination of drugs with EPL has the advantages of deeper penetration, higher stability which means longer retention time, better encapsulation efficiency and even better therapeutic effect than antitumor drugs solution alone, especially benefit to hydrophobic drugs with vitamin E succinate-grafted, PEGylated or thiolated and folic acid decorated EPL (Xu et al., 2017; Li Y. et al., 2020; Shi et al., 2020). Meanwhile, these modifications of EPL also improve the specificity of these antitumor drug system, show a pH-triggered or reductive-triggered drug release manner as synthesizing EPL with PEG or incorporate the disulfide bond into molecular structure, respectively. And the addition of some compounds such as folic acid could help EPL to activate folate receptor-mediated targeted endocytosis (Shi et al., 2020), which help further promote the specificity of these drug systems.

When tetraphenylethylene or acylated fluorescein is incorporated into the EPL-related drug system, it could show an aggregation-induced emission effect or a fluorescence that could be used in positron emission tomography treatment, bioimaging, or theranostic platform (Huang et al., 2015; Li Y. et al., 2020). EPL could also be associated with gene therapy, such as the formation of Ca^2+^/pDNA/polycationic nanocomposites, as a new stable gene vector, with the addition of adenosine triphosphate to improve transfection efficiency (Choi et al., 2018) or as a ligand to G-quadruplex, a secondary structure of DNA, with different binding modes in accordance with its target G-quadruplex's topologies (Marzano et al., 2020). By combination with these agents, the whole system could show better effectiveness and lower toxicity than injecting an equal dose of antitumor drugs alone and aid in locating the lesion sites. As a result, more studies are needed to use EPL in broadened clinical fields.

#### Other Drugs Delivery System

In addition to delivering antitumor drugs, EPL and its derivates are widely used as carriers of other drugs, especially proteins and peptides, which could be easily removed in the internal environment by effects like proteolysis or liver clearance. For example, when conjugating or electrostatic binding with β-cyclodextrin, the system could be a substantial candidate to be the carrier of bilirubin in islet implantation, with great biocompatibility and longer retention time (Yao et al., 2020), or as a broad-type protein carrier to overcome the gastrointestinal barrier, avoid digestion, and promote protein entry into the intestine (Niu et al., 2019). Moreover, when self-assembled by ionotropic complexation method, conjugates as EPL-sodium alginate showed an enhanced uptake of modeling antigen-bovine serum albumin with limited cytotoxicity and mild release. Thus, it could also potentially be used in vaccine delivery (Yuan et al., 2018).

In addition to the above applications, with a large number of amino groups on the side chains, EPL could be combined with more kinds of substances through covalent or non-covalent bonds and used in the treatment of many diseases. For example, it could be used to consist the triblock copolymer with polyethylene oxide and polypropylene oxide, which is the matrix of forming a thermo-responsive gel solution with rapid gelation. And the gel could enhance the release and uptake ratio of bone morphogenetic protein 4 and inhibit corneal neovascularization after eye injury (Xu et al., 2020). Also, the compounds composed of EPL combined with hyaluronic acid and parathyroid hormone-related protein could be used in forming multilayers as modification to titanium implants used in osteoporosis treatment, and enhance the local bone formation (Tang et al., 2020). They could also be synthesized as lipase-(ε-polylysine-heparin)-glassy carbon electrode to help detect triglycerides, one of the main biomarkers of cardiovascular diseases (Xu et al., 2018). Under some conditions, they could even show a special immune hemoglobin stimulation activity (Yuan et al., 2018).

The above findings showed that EPL has been widely used in the field of treatment, including that of several thorny diseases, and it could be used in the detection of blood routine examination. The achievement of this function mainly depends on the affinity of EPL to its target molecules or drugs, its biocompatibility and ability to avoid proteolysis then prolong the retention time, thus improving the efficiency of these drugs and making specific releases a reality. Although some contributions in medical treatment have been realized, more clinical trials are needed to make the use of EPL practical.

## Lysine-Based Dendrimer

### Structure and Synthesis

**Figure 3:**
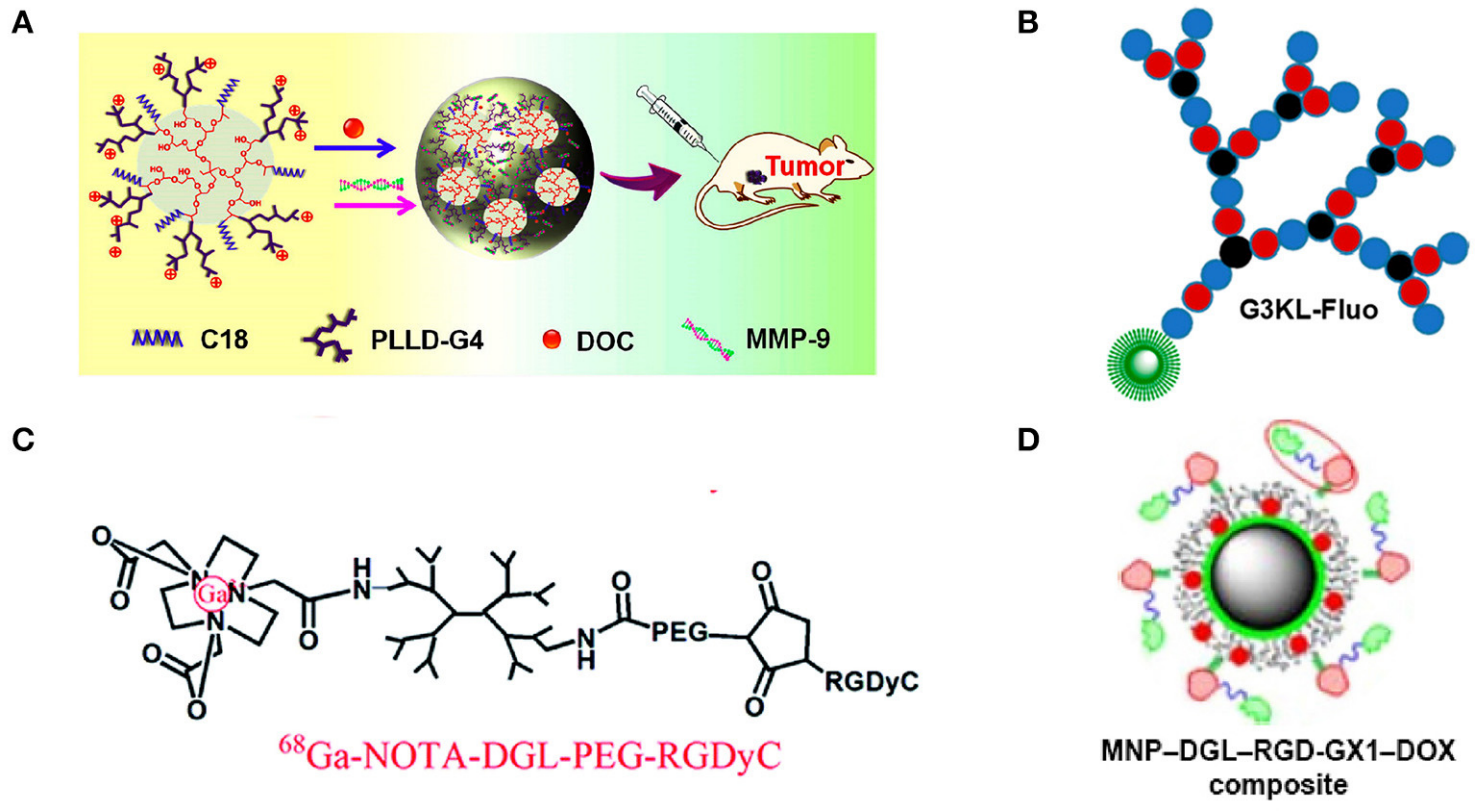
Illustrations to modification of lysine-based dendrimers: **(A)** Interior block [PLLD-G4, (Zhou et al., 2016)]; **(B)** Incorporation [G3KL-Fluo, (Gan et al., 2019)]; **(C)** Exterior modification [^68^Ga-NOVA-DGL-PEG-RGDyC, (Fang et al., 2020)]; **(D)** Combination [DGL-coated MNP, (Shen et al., 2017)].

Dendrimers are types of polymers with different generations of branches constructed in a dendritic form. Poly-L-lysine dendrimers (PLLDs) have attracted wide attention because of their great biocompatibility and numerous functional terminal amino groups. However, due to limitations, such as extremely strong electrostatic interaction in physiological environment, or for the expansion of the applications of this structure, modifications are needed. The four main types of modification for PLLDs could be summarized as interior blocks (also called dendrigrafts), incorporation (normally called lysine-based dendrimer), exterior modification, and modified in combination strategy (Figure 3). As all of them are based on the PLL backbone or form a dendrimer branched by lysine monomer, they were all referred to as LBD in this review.

Different structures endow polymers with different properties, and they are synthesized by different methods. For PLLDs under the third generation, the synthetic methods are quite mature, which could be solid phase polypeptide synthesis (Shen et al., 2017; Fang et al., 2020) or synthesized with monomers protected by groups, such as tert-butoxycarbonyl (Boc), fluorene methoxycarbonyl (Fmoc), and carbobenzoxy. For example, Boc-Lys(Boc)-OH or Boc-Lys(Boc)-OSu could be used as the monomer to synthesize PLLDs with an initial core compound, such as butanediamine, and increase its generation step-by-step. Some researchers also synthesized PLLDs through divergence–convergence method (Wu C. et al., 2018), which considerably improved the synthesis efficiency.

The highest generation of PLLD could reach five thus far (Mehta et al., 2018), and by connecting polyethylene glycol (PEG) to the exterior of fourth generation PLLD, a fifth pseudo-generation PEGylated PLLD could be synthesized (Haque et al., 2017). Unlike polyetherimide, which could form an intact symmetry structure then be totally modified (Walsh et al., 2018, 2019), increased defects in the structure of PLLDs could be found with the increase in generation (Weiyue et al., 2020). The existence of these defects may be derived from the steric hindrance or the different lengths of the α- and ε-amino branches formed on the PLLD backbone that hides some of the amino functional groups inside without participating in the next steps of polymerization. Meanwhile, both difficult purification and high possibility of forming assembly precipitation hinder the application of PLLDs.

**Figure 4:**
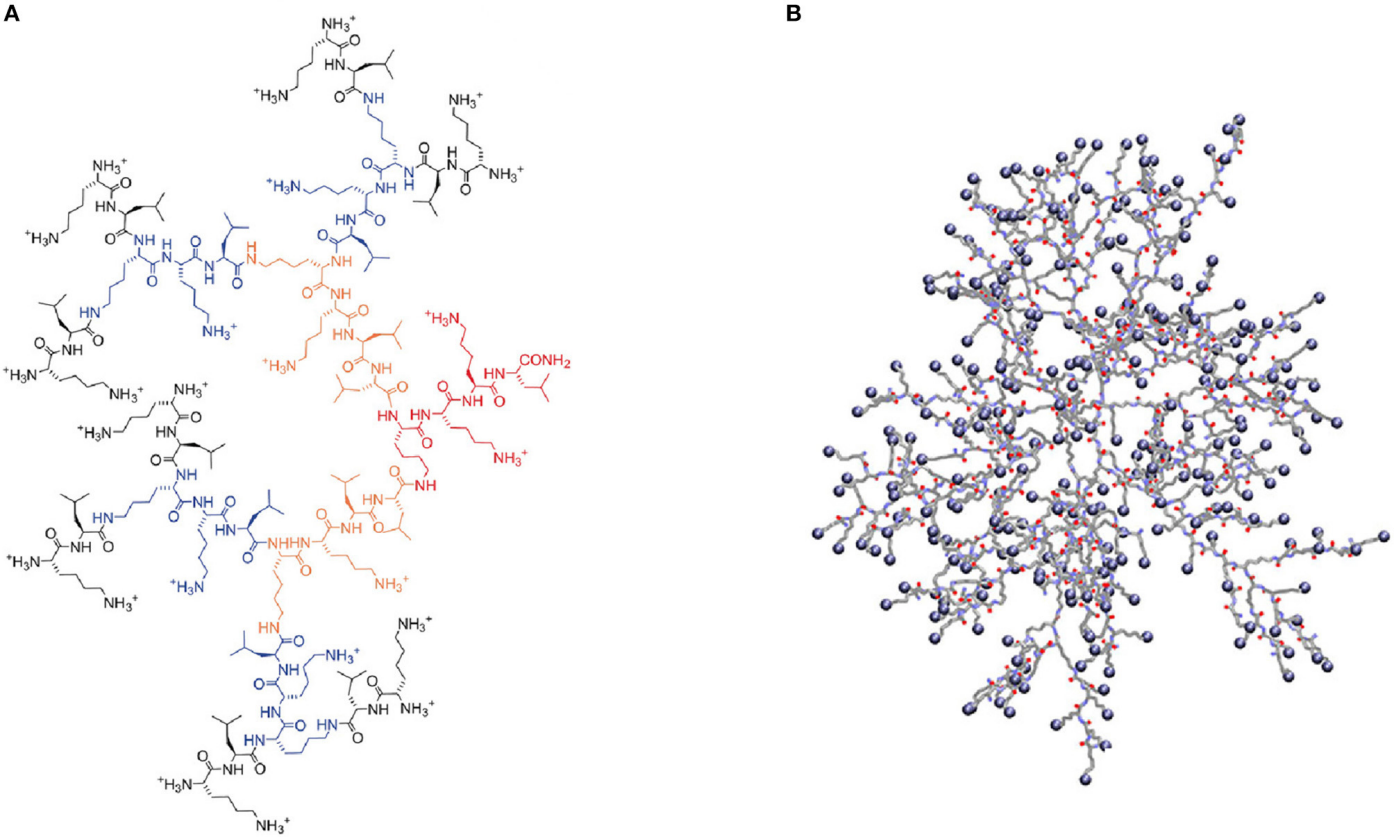
**(A)** Chemical structure of G3KL (Siriwardena et al., 2018a); **(B)** Snapshot of G4 polylysine dendrimer in molecular dynamic simulation (Francoia et al., 2017).

In practical application, PLLDs are hardly used without modification for its cytotoxicity by its numerous and dense cationic ${\text{-NH}}_{3}^{+}$ on the molecular surface when existing in physiological environment. Thus, doing some exterior modifications, like PEG coating (Haque et al., 2017), is normal to reduce the surface charges; or changing the composition in the middle, namely incorporation modification, thus making a loosened distribution of ${\text{-NH}}_{3}^{+}$ and forming a new structure, such as G3KL (Figure 4A), which is a third-generation dendrimer consisting of lysine as branch monomer and two leucine monomers incorporating inside (Gan et al., 2019). Moreover, PLLDs could be used as a modifier coating on some magnetic particles (Shen et al., 2017; Lataifeh and Kraatz, 2019) or linking with some block polymers enlarging LBD's zone area (Zhou et al., 2016). Considering these different types of modification, the structure of LBD is complicated and suitable for using in different applications.

In addition to traditional chemical synthesis and characterization, molecular dynamic simulation techniques have been introduced into functional analysis to improve the efficiency of LBD synthesis (Francoia et al., 2017; Okrugin et al., 2018). Along with enumerating virtual libraries and chemical spatial analysis to find the optimal structure, the design process can also be speeded up (Siriwardena et al., 2018a).

Several factors may affect the performance of LBD, including generation, size, topology, and modification strategies. As the generations increase, LBD could improve the retention time and loading effects (Li et al., 2016; Wu C. et al., 2018) but may also result in increased cytotoxicity and decreased antiviral effect (Weiyue et al., 2020). They found that the size of polylysine dendrigraft with the same molecular weight and density depends on its side chain topology. However, regardless of the topology, all of them have the same nearly spherical morphology (Okrugin et al., 2018). Molecular dynamic simulation of microsecond showed that PLLDs could form plentiful conformation without folding (Francoia et al., 2017), thereby demonstrating the multifunctional potential of LBD. Combined with different modifications and conjugated with other compounds, LBD could undoubtedly be modified to suit various applications.

### Advanced Applications of LBD in Biopharmaceuticals

Similar to EPL-related applications, LBD-related studies have focused on applications such as diagnosis, gene transfection, and antitumor auxiliaries in recent years (Feng et al., 2019; Ge et al., 2019). LBD is usually used in drug delivery systems with modification by other compounds because of its large self-assembly tendency and large capacity interior (Li et al., 2016; Kim et al., 2018; Wu C. et al., 2018; Agazzi et al., 2020). Great biocompatibility and biodegradable activity also expand its application possibility in the field of biopharmaceutics. The rich and controllable terminal groups of different generations of LBD provides convenience to the modification and makes the application of the complexes further expanded.

#### Antimicrobial, Antifungal and Antiviral Agents

With large amounts of amino groups in the terminal, which exhibited strong cationic effects in psychological environments, LBD shows an immense ability of bacterium inhibition. Among which, G3KL, a dendrimer formed by lysine and leucine, has been widely proven to have considerably antimicrobial ability to Gram-negative bacteria, even multidrug-resistant clinical isolates. The antibacterial effects work mainly with the mechanism as outer membrane permeation and disruption, while internal membrane depolarizing and endotoxin lipopolysaccharide neutralizing also contribute (Pires et al., 2015; Siriwardena et al., 2018a,b; Gan et al., 2019; Ben Jeddou et al., 2020). Apart from avoiding infection, the compound has a pro-angiogenic activity in burn wound healing by speeding up the recovery (Abdel-Sayed et al., 2016). Besides effectively inhibiting *Candidiasis* with Trp-rich structure (Zielinska et al., 2015), LBD exhibited a synergistic antifungal property when conjugated with N^3^-(4-methoxyfumaroyl)-(S)-2,3-diamino-propanoic acid, an antifungal agent inhibiting GlcN-6-P synthase, and the drug system even found to be effective to fluconazole-resistant strains, especially in YNB-SA minimal medium (Stolarska et al., 2020). Meanwhile, LBD could show a more potent antihuman immunodeficiency virus activity at the moderate molecular distance when modified by cellobiose with sulfated treatment to its second generation (Weiyue et al., 2020), thus proving its potential to be used as an antiviral agent.

However, LBD and its derivatives are not as suitable in the antibacterial field compared with EPL because their cation distribution is too dense and may cause obvious toxicity. Therefore, to improve its ability as high efficiency with low toxicity, medical safety index was used by dividing the minimum inhibition concentration by half hemolytic concentration in some cases (Grimsey et al., 2020). Modifications are needed to neutralize some of the cationic branches and disperse the concentrated positive charges, including lengthening the branches (Gan et al., 2019) and partial conjugation with polysaccharide (Weiyue et al., 2020).

#### Drugs Delivery System

Similar with EPL, applications as antitumor drug delivery vehicles largely accounted in LBD studies (Li et al., 2016; Shen et al., 2017; Zhang et al., 2017). With a large capacity and vast of functional groups, LBD could load various drugs, including growth factors (such as nerve growth factors) that stimulate neuronal length and number growth (Kim et al., 2018), enzymes (such as nattokinase) that could be applied in thrombolysis (Wu C. et al., 2018), and antitumor drugs (such as gemcitabine) assisting with the inhibition of tumor growth (Zhang et al., 2017). It could also endow hydrophobic drugs with enhanced cell membrane permeability and faster internalization (Li et al., 2016) and assist with the drug penetration of the blood–brain barrier (Kim et al., 2018). The performance of drugs encapsuled in these carrier systems could even be more reliable than the drugs alone under the same dose of solution (Zhang et al., 2017). Therefore, finding a suitable carrier is necessary to avoid hard uptake, instability, or any reason that may affect the performance of drugs in the internal environment.

**Figure 5:**
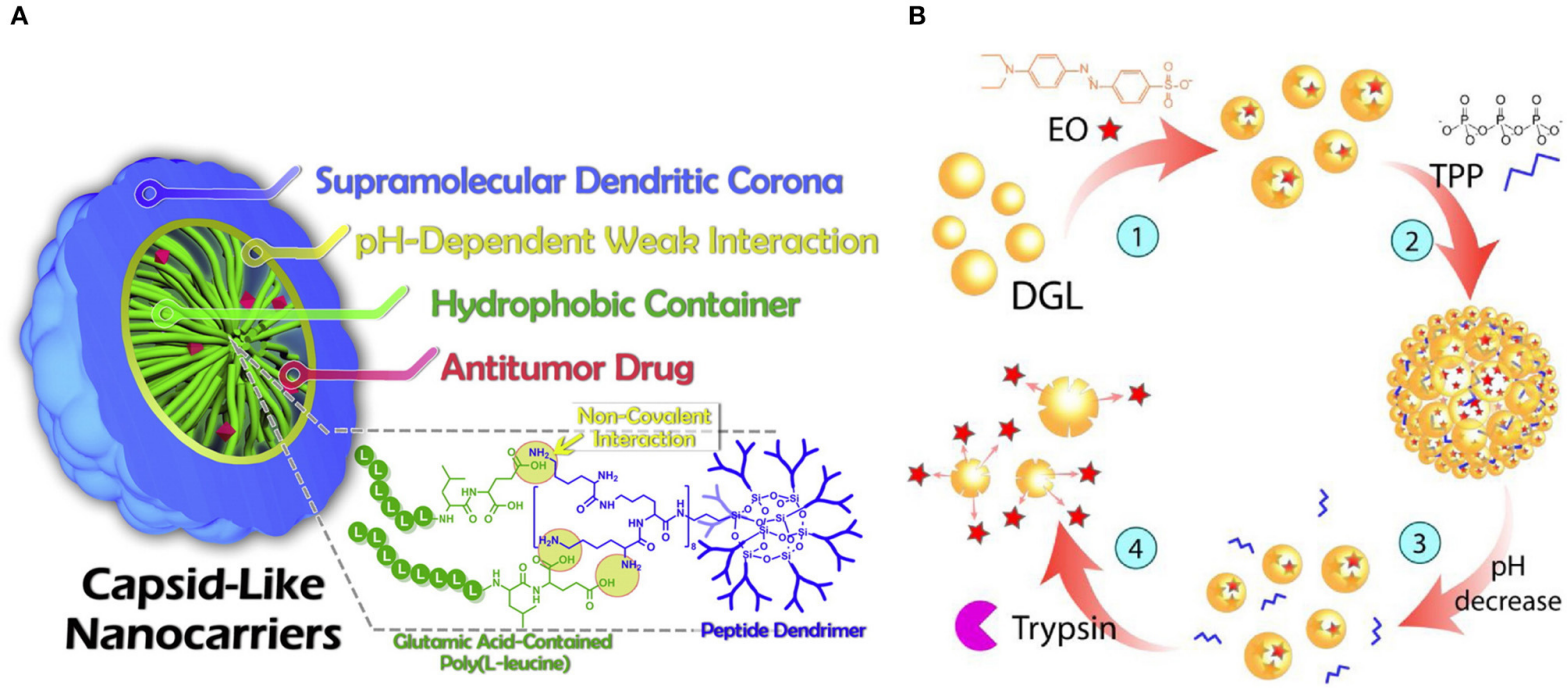
Illustrations of two types of LBD conjugates for drug delivery system. **(A)** Non-covalent capsid-like nanocarriers (Li et al., 2016); **(B)** Drug delivery system with multistage stimuli responses (Agazzi et al., 2020).

One of the aspects that needs to be considered in designing the carrier system and improving drug efficiency is the combining or conjugating stability. The driving force between drugs and LBD include electronic interaction, hydrogen bond (Figure 5A), and van der Waals force (Li et al., 2016; Wu C. et al., 2018). Sustainable releases at low levels and for longer cycles are urgently needed to reduce the side effects of drugs at high concentrations. However, the needs satisfaction depend largely on LBD biodegradation or broken structures as a response to certain stimuli, such as pH (Li et al., 2016), temperature (Kim et al., 2018), and enzymes (Zhang et al., 2017); or combination with order. For example, encapsulating drug DOX and curcumin for a sustained release (Agazzi et al., 2020). Firstly, size changed responding to pH, then partial proteolysis of the PLLD carrier was stimulated by trypsin, with DOX and curcumin released finally (Figure 5B). Different stress responses depend on conjugated molecules of the delivery system, which also endows the system with site-specific release patterns, thus overcoming the disadvantages that pure PLLD and antitumor drugs may have, such as poor specificity and uncontrolled distribution (Li et al., 2016; Shen et al., 2017; Zhang et al., 2017; Agazzi et al., 2020). Theses disadvantages may exhibit as low efficiency and same level of toxicity to normal tissues as tumors, leading to poor prognosis.

#### Adjuvants of Gene Therapy

The application of LBD as a gene delivery vehicle could be strictly regarded as part of the drug delivery, but the loaded drug specifically points to nucleotides, especially small interfering ribonucleic acid (siRNA). As a cationic carrier in physiological environment, which does a favor to the interaction with nucleic acid without a covalent bond, LBD has shown great potential as a carrier for gene therapy. Meanwhile, as a dense group of distribution exists outside, LBD carriers protect the loaded ribonucleic acid from immune responses and nuclease degradation (Han et al., 2018). This non-viral system shows many advantages, including higher loading capacity, less inflammation potential, lower insertion mutagenic induction potential, cheaper scale, and easier functionalization, than the viral system (Walsh et al., 2018; Alazzo et al., 2019; Gorzkiewicz et al., 2020a). All these advantages make this non-viral system attract increasing attention.

Incorporating two amino acids into the backbone of PLLD, such as forming G3KL, not only exhibited an antimicrobial activity but also changed the performance of the carrier system, which could help promote the transfection efficiency and specifically improve the cytotoxicity to the cancerous cells, such as Hela cells. It even showed better performance than commercial merchandise lipofectamine (Gorzkiewicz et al., 2020a,b). The main mechanism of these phenomena has been demonstrated as inducing apoptosis in the beginning while being genotoxic with longer incubation (Gorzkiewicz et al., 2020a). In addition, cell metabolite analysis showed that hyperbranched polycation–DNA complexes made an alteration in energy metabolism and consequently influenced the redox homeostasis of cells (Alazzo et al., 2019).

A correlation exists between toxicity and transfection efficiency (Alazzo et al., 2019) but this does not seem to be the case with sugar-functionalized PLL synergistic compounds (Han et al., 2018), which showed great affinity for loading siRNA, less cytotoxicity and maintained transfection efficiency as the original PLL modified by stearylamine. Surprisingly, it could even knock down some disease-related genes. Therefore, further research is needed on the relationship between its toxicity and gene transfection efficiency to help design safer drugs.

A co-transmission of antitumor drugs and targeted genes is required to inhibit drug resistance, such as proliferation of nuclear antigen expression; reduce the adverse effects of drugs; and enhance the synergistic effects. It could be achieved by cyclodextrin-PLLD vector system (Liu et al., 2017) or micelles formed by amphiphilic molecules connecting nucleic acids and antitumor drugs at different sites of the vector (Zhou et al., 2016). By mediating concentrations thus prolonging the retention time, the former effectively inhibits nasopharyngeal carcinoma tumors, and seems to be safer to brain and easier to synthesize without an uncontrollable micellization process (Liu et al., 2017). However, it also exhibited limited specificity and dramatically increased DOX concentrations in the liver and kidney, and the latter could address these issues together with an improved blood compatibility. Compared with the comparative matrix metalloproteinase-9 protein expression inhibition and transfection efficiency, the toxicity is negligible and induces more apoptosis in breast cancer cells than using DOX or matrix metalloproteinase-9 alone (Zhou et al., 2016).

#### Imagining Agents and Adjuvants to Tumor Therapies

Imaging techniques and agents are urgently needed for precise excision without tumor recurrences and poor prognosis and for early diagnosis and treatment. Tripeptide arginine–glycine–aspartic (RGD)-related compounds, such as c(RGDyK) peptide (Feng et al., 2019), RGDyC (Fang et al., 2020), and synergistic compound RGD-GX1 (Shen et al., 2017), have been commonly used as specific α*νβ*3-integrin ligands in drug systems to enhance target efficiency, which are related to the formation of tumor neovascular and widely found overexpressed in tumor tissue surroundings. With combination with near-infrared ray (NIR) fluorescent or ^68^Ga, the system could be used in applications such as near-infrared or positron emission tomography imaging, respectively, with clear imaging and high tumor-to-normal tissue intensity ratio for the former system and high uptake speeds, great stability, quick clearance, and slight cytotoxicity for the latter (Feng et al., 2019; Fang et al., 2020). It could also be used in MRI as an adjuvant agent and as an antitumor drug delivery system for DOX for earlier diagnosis and therapy for hepatocellular carcinoma by assembling layer-by-layer to form the complex RGD-GX1 heterogeneous dimer peptide-conjugated dendrigraft PLL–magnetic nanoparticle (Shen et al., 2017). Experimental results showed that the targeting ability of this drug system has been substantially enhanced while reducing the toxicity of DOX to normal tissues.

PDT and PTT have been commonly used in the antitumor therapy in recent years. Plasmonic gold nanoparticles with hyperbranched PLL deposited has formed in aqueous solution to be used as a PTT auxiliary agent, which exhibited the ability to quickly elevate the solution by 23.1°C after 5 min of NIR laser irradiation with great photostability and high photothermal conversion efficiency (Ge et al., 2019). These results proved the ability of LBD to be used in more medical therapies as a modifier to other medicines.

Besides all of the above, branched PLL could be used as an *in-situ* temple and catalyst of bio-silicification to form branched PLL/silica hybrid particles with diverse morphologies. The achievement of this function relies on the abundant hydrogen bonds of branched PLL to self-assemble and the ability to conform transition (Min et al., 2018). Therefore, LBD could be used in imaging identification, bio-silicification, or other aspects, not only as antimicrobial or antiviral drug carriers of these drugs, proteins, and nucleic acids but also as adjuvants, modifiers, temples, or catalysts for certain drugs. Furthermore, more application fields could be found if the large and controllable number of functional groups in LBD could be properly utilized.

## Challenges and Future

Without structural and performance evaluation, judging the success of designing or synthesizing a product is impossible. Therefore, many techniques, such as high content screening and nanoparticle tracking analysis, have been incorporated into the evaluation process of these polymeric complexes. With these instruments and some digital simulation strategies, the efficiency of the design process could be increased. Therefore, with several years' development, the synthesis and application strategies of EPL and LBD have further matured, which contributes to the development of clinical medicine and brings hope to those targeting patients.

**Figure 6:**
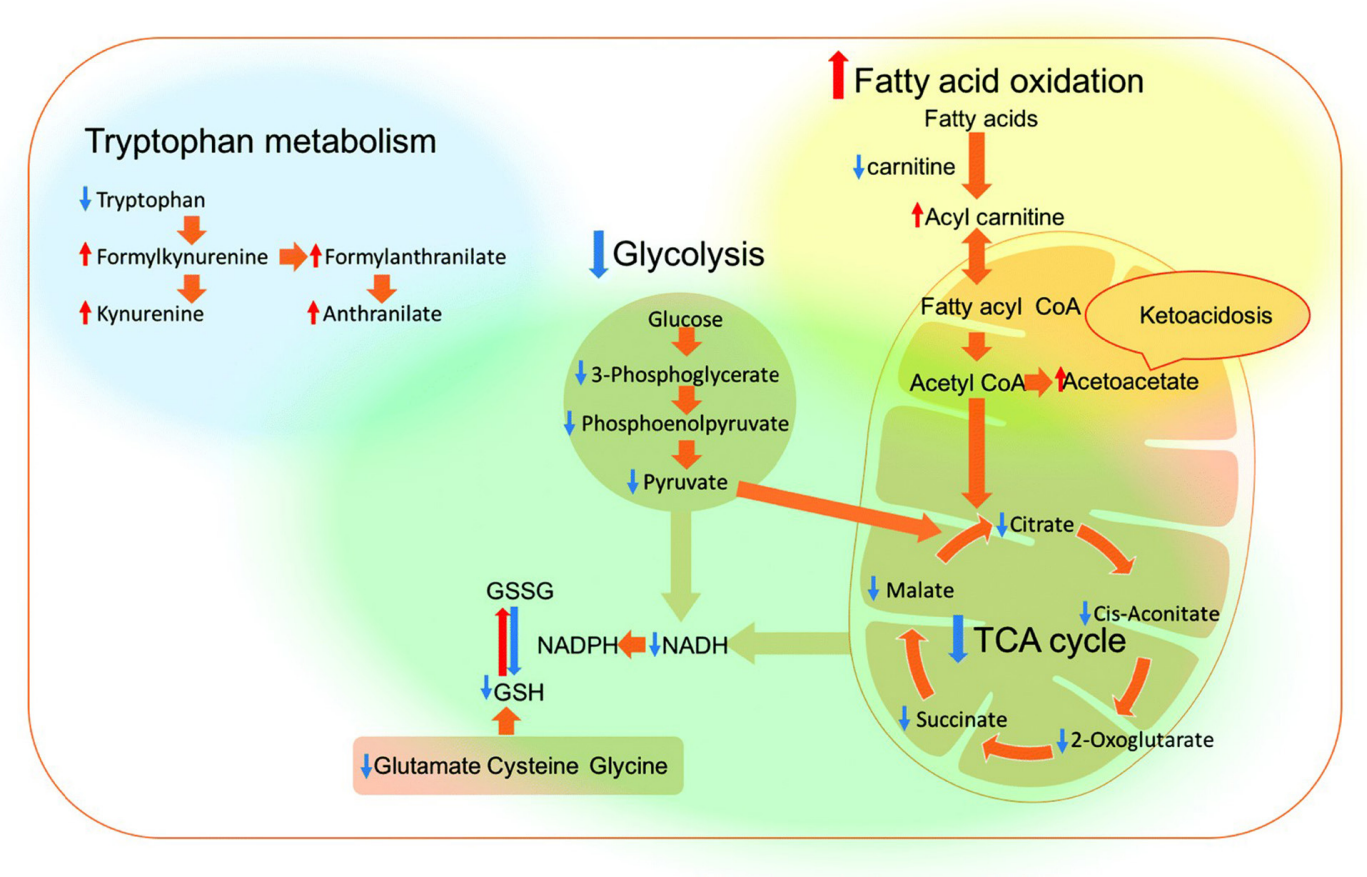
Overview of the affected metabolic pathways in the A549 and H1299 cells after treatment with the DNA conjugating LBD polyplexes (Alazzo et al., 2019).

Furthermore, the pharmacodynamics and pharmacokinetics of the drug system are essential in the drug designing. However, the working mechanism of EPL and LBD-related drug systems have not been clear enough. Thus, increased attention should be paid in exploring more therapeutic mechanisms, such as affecting the tricarboxylic acid cycle and the redox state of metabolites (Alazzo et al., 2019) (Figure 6), influencing protein secretion (Walsh et al., 2018), or activating the expression of related gene (Li S. et al., 2020). Meanwhile, the uptake pathway (Walsh et al., 2018), and removal strategies of tissues such as liver and kidney should also be concerned, which reflects as drug effectiveness, retention time or other performances. Not only uptake efficiency, the topic also cares about properties like the specificity to the target and cytotoxicity, which needs better comprehensions to the differences between tumor cells and normal ones although with similar cell structures. In some theses, the carrier's cytotoxicity with cancerous cells were tested first, ensuring the toxicity is not from the outer shell but from the drugs loaded internally (Kim et al., 2018), which could be released and be taken in a specific tumorous condition, such as acidic surrounding, rich-glutathione, overexpressed enzymes and hyperpyrexia (Li et al., 2016; Kim et al., 2018; Mehta et al., 2018; Shi et al., 2020). Therefore, studies about the differences between the surroundings of normal and cancerous cells are also essential for designing new drugs with specifically higher response.

Besides mentioned above, suitable size and structure are essential in drug design. However, conflicts regarding the optimal size exist. In fact, this may depend on the functional purpose of the design. Small particles were found to promote the penetration, while large particles endow drugs with longer retention time (Li et al., 2016). As for those PEGylated LBD, intermediated size, which means longer PEG chain with lower generation PLL core or higher generation core with shorter PEG lateral chain, demonstrated to exhibit superior in those anticancer activity (Mehta et al., 2018). Furthermore, the assembling structure and strategies could influence the drug application. As supermolecules, self-assembled dendrimers showed an improved performance in a facile procedure of manufacturing with low toxicity and without vanishing the function of interior particles (Li et al., 2016; Liu et al., 2017). As mentioned in the above caption, strategies to maintain the integrity of high-generation lysine dendrimers and balance the toxicity and antibacterial activity are also necessary in drug design.

Last but not least, the expenses and production scale should also be considered when designing these drug systems. Although some of the products have shown a comparable excellent performance, the expensive and intricate synthesizing procedures hamper their expansion to the large-scale production and clinic application. The different efficacy results of the same drug are widespread in *in-vitro* and *in-vivo* experiments (Zhou et al., 2016), as is the way between animal experiments and clinical use. Why do these difference exist and how to avoid them to make the valuable research products into practically applications are indeed worthy of explorations. And it may be one of the reasons why drugs need to be verified for a long period, from cells to various animals, and even require three periods of clinical trials.

Although the synthesis and application of EPL and LBD continue to have many challenges to face, with the combination of some advanced analytical and simulation techniques, ingenious machines, and fine experimental strategy design coupled with clearer mechanistic studies, EPL- and LBD-related drug systems are considered promising for the search or design of novel and efficient drugs in the treatment of those troublesome diseases.

## Author Contributions

SC and SH make up the draft of the manuscript. CZ and YL supervised and revised it. All authors contributed to the article and approved the submitted version.

## Conflict of Interest

The authors declare that the research was conducted in the absence of any commercial or financial relationships that could be construed as a potential conflict of interest.
